# Distinct Imaging Features of Peripheral Nerve Sheath Tumours in *NF2*-Related Schwannomatosis: A Case Report

**DOI:** 10.1155/crnm/6923539

**Published:** 2025-10-09

**Authors:** Küpper Hanna, Roggia Cristiana, Winter Natalie, Grimm Alexander, Nägele Thomas, Rosewich Hendrik, Schuhmann Martin Ulrich

**Affiliations:** ^1^Department of Neuropaediatrics, University Hospital Tübingen, Tübingen, Germany; ^2^Institute of Medical Genetics and Applied Genomics University Hospital Tübingen, Tübingen, Germany; ^3^Department of Neurology, University Hospital Tübingen, Tübingen, Germany; ^4^Department of Diagnostic and Interventional Neuroradiology, University Hospital Tübingen, Tübingen, Germany; ^5^Department of Neuropaediatrics, University Hospital Göttingen, Göttingen, Germany; ^6^German Center for Child and Adolescent Health (DZKJ), Partner Site Göttingen, Göttingen, Germany; ^7^Division of Paediatric Neurosurgery, Department of Neurosurgery, University Hospital Tübingen, Tübingen, Germany

**Keywords:** nerve ultrasound, NF2-related schwannomatosis, perineurioma, peripheral nerve sheath tumours (PNST), schwannoma

## Abstract

**Introduction:**

Peripheral nerve sheath tumours (PNST) are an important feature of the *NF2*-related schwannomatosis. These constitute primarily schwannomas which are characterized as nodular, solitary benign tumours of single fascicles. We here describe a pronounced, extensive, multifascicular enlargement of the right ulnar nerve and bilateral brachial plexus in a teenage patient with *NF2*-related schwannomatosis. Neither their imaging nor the clinical characterization were consistent with classic schwannomas.

**Methods:**

Within a multimodal work-up, detailed and standardized high-resolution nerve ultrasound was correlated to clinical, electroneurographic, and MRI findings. Follow-up data over 2 years were provided.

**Results:**

The clinical presentation of a slowly progressive, predominantly motor axonal neuropathy of the right ulnar nerve prompted a comprehensive nerve ultrasound examination, initiated by this clinically and neurophysiologically defined “sentinel” finding. Ultrasound imaging revealed an extensive, fusiform enlargement of the right ulnar nerve, as well as bilateral involvement of the brachial plexus, the latter without corresponding clinical symptoms. Subsequent genetic analysis confirmed the diagnosis of NF2-related schwannomatosis.

**Discussion:**

Signs of a mononeuropathy are a frequent manifestation of *NF2*-related schwannomatosis in childhood. The here presented nerve imaging characteristics, however, did not correspond to classic schwannomas. The fusiform, long-extended, multifascicular enlargement with mixed hyper- and hypoechogenic components on ultrasound, as well as markedly T2-hyperintense and T1-isointense appearance with diffuse gadolinium enhancement on MRI, is consistent with perineurioma. These mesenchymal nerve tumours have rarely been described in *NF2*-related schwannomatosis. This association is further supported by the present case and highlights the necessity for additional peripheral nerve imaging studies in NF2-related schwannomatosis, including the application of high-resolution ultrasound.

## 1. Introduction

The *NF2*-related schwannomatosis is a rare autosomal dominant tumour predisposition disorder, linked to the neurofibromatosis type 2 (*NF2*) tumour suppressor gene on chromosome 22q [1, 2]. It has a broad phenotypic spectrum, affecting the nervous system, eyes and skin [3, 4]. Hearing loss is the typical presentation in adulthood, due to bilateral vestibular schwannoma. When presenting in childhood, signs of a mononeuropathy, skin tumours or ophthalmologic pathologies are frequent [3]. Tumour entities of the cranial nerves include the characteristic bilateral vestibular schwannomas. Meningioma, glioma, astrocytoma and spinal ependymoma are other manifestations in the central nervous system [4].

Peripheral nerve sheath tumours (PNST), primarily schwannomas, are a further important hallmark of this syndrome [5]. Schwannomas are benign PNST which present as subcutaneous nodules, originating from cutaneous sensory nerves, as well as from major peripheral nerves [6]. The distinctive presentation is a slowly growing mass, with possible symptoms of nerve compression, typically sensory (paraesthesia, numbness, and radiating pain). Motor neuropathic symptoms are occasionally described [7]. Morphologically, schwannomas are solitary spindle cell tumours, with a cut surface of a fibrous capsule [8]. Only few nerve ultrasound studies of *NF2*-related schwannomatosis have been published to date [9, 10], examining solely adults. They describe classic schwannomas as oval-shaped, homogeneous hypo- to isoechoic nodules of single fascicles, displacing normal fascicles, with well-defined margins. On MRI [11], they are characterized as T2-hyperintense, nodular “macro- and microlesions” of peripheral nerves.

However, the here presented clinical as well as imaging characteristics of a teenage girl carrying a pathogenic variant in the *NF2*-gene with an extensive, multifascicular enlargement of the right ulnar nerve and parts of the brachial plexus do not correspond to classic schwannomas. For differential diagnosis, a detailed analysis of the here presented imaging data is correlated to reports of *NF2*-related schwannomatosis in the literature.

## 2. Methods

Ultrasound of peripheral nerves and muscles was conducted with a high-resolution (18 MHz) broad band linear probe (Canon *Aplio a, Canon Medical Systems GmbH, Germany*) with standardized ultrasound settings on yearly follow-up examinations over a period of 2 years. Cross-sectional area (CSA) and diameter of peripheral nerves and cervical roots were determined and correlated to detailed clinical examinations as well as focussed nerve conduction studies. Contrast-enhanced MRI of the lower right arm and bilateral brachial plexus was performed once, of the brain and spinal cord twice. Patient consent was obtained.

## 3. Results

At the age of 10 years, she presented with a motor predominant deficit of the right ulnar nerve which, retrospectively, had slowly developed over 3 years. There was pronounced weakness (Medical Research Council (MRC) Scale for Muscle Strength 3/5) for the abduction of the right little and index finger, with significant atrophy of the abductor digiti minimi and interosseus dorsalis muscles. The sensation for touch was mildly reduced in the area of the right dorsal ulnar branch. On nerve conduction study, signs of a predominantly motor axonal neuropathy were depicted for the right ulnar nerve, with normal findings for the left ulnar and right median nerve. Follow-up over 2 years was stable (Supporting table 1).

**Figure 1:**
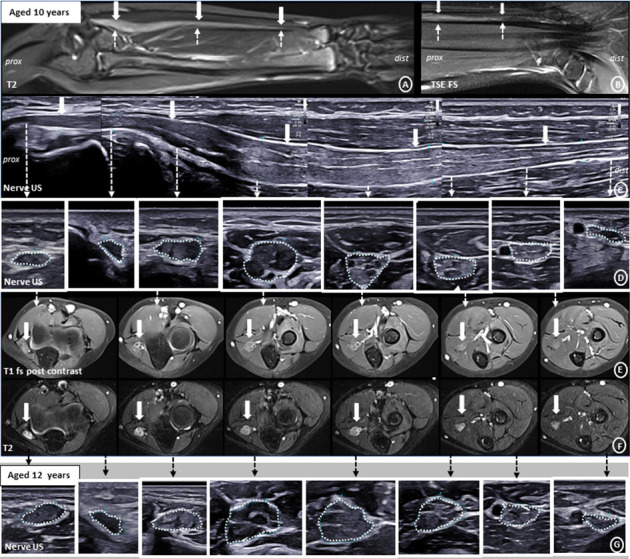
Imaging characteristics of the significant, long-extended enlargement of the right ulnar nerve throughout its entire course from the distal upper to distal lower arm. On coronal T2-weighted MRI (A), coronal Turbo-Spin-Echo fat-suppression MRI (B) and axial T2-weighted MRI (F), a marked hyperintensity of the entire ulnar nerve is visible. Sagittal (C) and axial (D) high-resolution nerve ultrasound depicts the pronounced fusiform enlargement of the entire nerve in the forearm, with multifascicular involvement, predominantly hypoechogenic, but partly inhomogeneous with hyperechogenic components. See also Videos 1–5 (Supporting Information) for a detailed documentation. Markedly reduced fascicular differentiation (“blurry” appearance) of the ulnar nerve in the proximal forearm (D) which becomes more pronounced in the follow-up after 2 years (G). Signs of contrast enhancement, especially in the perineurium, on axial T1-weighted MRI (E).

**Figure 2:**
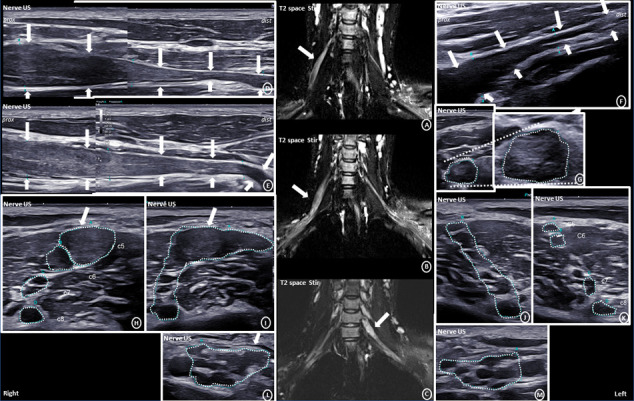
Imaging characteristics of the significant, long-extended enlargement of the right cervical nerve root C5 and C6 in its distal course and transition to super truncus with marked hyperintensity on coronal T2 Space Stir (A) (B) and predominantly hypoechogenic appearance of fusiform enlargement on longitudinal nerve ultrasound (D) (E). Multinodular, hypoechogenic appearance on axial imaging of the plexus in the interscalene (H) (I) and supraclavicular (L) region. See also Videos 6-7 (Supporting Information) for a detailed documentation. Significant, long-extended enlargement of the left cervical nerve root C7 in its proximal part with marked hyperintensity on coronal T2 Space Stir (C) and predominantly hypoechogenic appearance of fusiform enlargement on longitudinal (F) and high-resolution axial nerve ultrasound of the proximal C7 root (G). Normal ultrasound appearance of the left brachial plexus in the interscalene (H) (I) and supraclavicular (L) region.

On nerve ultrasound, there was pronounced long-extended enlargement of the right ulnar nerve, throughout its course from the elbow to the wrist, with a maximum CSA of 38 mm^2^ in the proximal forearm (Figure 1(D)). The enlargement was fusiform, multifascicular, mixed hypo- and hyperechogenic, without signs of calcifications. The fascicular pattern was preserved. However, at the site of maximum enlargement, fascicular differentiation was reduced. The outer margins of the ulnar nerve were well defined. Morphological appearance of the nerve proximal and distal from the enlargement was normal, and there was gradual tapering of the enlargement. On Doppler sonography, there was no increased vascularization. A similar, but more multinodular pattern of enlargement, was depicted in the right cervical nerve roots C5 and C6 as well as left C7 (Figure 2). On ultrasound follow-up after one and 2 years, the fascicular differentiation within the maximally enlarged nerve segments appeared further reduced, while the absolute extent of nerve enlargement seemed comparable (Figure 1(G)).

**Figure 3:**
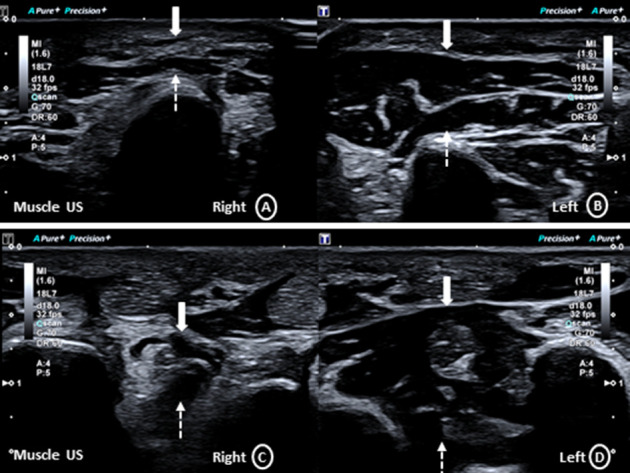
Axial muscle ultrasound of the right (A) and left (B) hypothenar as well as right (C) and left (D) interdigital muscles, with marked hyperechogenic atrophy of the abductor digiti minimi and dorsal interosseus muscles on the right side.

Muscle ultrasound depicted a hyperechogenic atrophy of the right abductor digiti minimi and dorsal interosseus muscles (Figure 3). Nerve ultrasound screening of other peripheral nerves was normal apart from some hypoechogenic, accentuated fascicles (Supporting table 2).

On MRI, the right ulnar nerve appeared T1-isointense, markedly T2-hyperintense and showed diffuse, moderate to marked contrast enhancement (T1) (Figures 1(A), 1(B), 1(E)). Laboratory work-up for inflammatory neuropathies revealed no significant abnormalities. After 2 cycles of intravenous immunoglobulin (2 g/kg), there was no significant clinical or imaging improvement. Subsequent genetic analysis, initiated originally for the differential of a rare hereditary neuropathy, revealed a disease defining pathogenic splice variant (c.1123-2A > G, p.?) in the *NF2* gene. Detection of small nodular schwannomas in the vestibular (3 × 2 mm) and mandibular nerve (4 × 3 mm) (Supporting Figure 1) on subsequent brain MRI confirmed the diagnosis of an *NF2*-related schwannomatosis [5]. Follow-up brain MRI after 1.5 years showed no significant change. Functional testing of the vestibulocochlear nerve, ophthalmological evaluation and spinal MRI were normal. MRI of the brachial plexus demonstrated a small additional enlargement of the left cervical root C4. The enlargements were T2-hyperintense, slightly inhomogeneous, with mild diffuse contrast enhancement.

## 4. Discussion

This is a case report of a rare combination of different nerve sheath tumours in a teenage patient with genetically confirmed *NF2*-related schwannomatosis. Specific findings of the ulnar nerve were highly suggestive of a perineurioma [12–16], i.e., the history of a teenage-onset, slowly evolving, painless, predominantly motor axonal mononeuropathy, with weakness and muscle atrophy, and, on imaging, a long-extended, fusiform, multifascicular nerve enlargement, with proximal and distal tapering, in combination with a T2-hyper- and T1-isointensity as well as a marked gadolinium enhancement on MRI. The here described nerve ultrasound and MRI features correspond closely to previous imaging descriptions in 6 histologically confirmed perineurioma cases with a similar history [17, 18], thus making other differential diagnoses very unlikely. Rarely, perineurioma has been described histologically in *NF2*-related schwannomatosis [12], as well as mutations in the *NF2* gene in histogenetic analyses of perineurioma [2]. The intraneural perineurioma is a benign PNST, with common locations in major peripheral nerves and brachial plexus. First described by Imaginarion in 1964 [19], the anecdotal connotation as a localized hypertrophic (mono)neuropathy (“dense swollen cord” [17]) or “interstitial hypertrophic neuritis” [19] as well as the Perineurioma Diagnostic Criteria [20] meet the here described characteristics [21]. To date, an epineurectomy and fascicular biopsy for further diagnostic and therapeutic purposes have not been conducted in this teenage patient, due to her long-standing stable clinical and electroneurographic course, but previewed in case of any change within the close monitoring.

The findings of the here described vestibular and mandibular nerves are, in contrast, characteristic of classic schwannomas [9–11], due to their nodular, homogenous enlargement and, to date, asymptomatic status. By comparison, the multinodular to diffusely thickened appearance [22] of the bilateral brachial plexus on ultrasound (supporting video 6-7) as well as the slightly inhomogeneous pattern with only mild diffuse contrast enhancement on MRI, without any clinical correlate, could be consistent with plexiform schwannomas. First characterized by Masson in 1970 [23], these have been described as benign variants in *NF2*-related schwannomatosis [1, 24]. However, their localization in the plexus is characterized as rare [25] and the morphological pattern often as irregular inhomogeneous masses [22], occasionally including degenerative changes (haemorrhage, cyst formation and dystrophic calcifications) which is not present here. Hybrid nerve sheath tumour (HNST) [26] in *NF2*-related schwannomatosis has, on the other hand, to date only been described for the combination of schwannoma and neurofibroma [27], not perineurioma and plexiform schwannoma. No characteristic features, though, of classic neurofibroma (small, hypoechogenic, and nodular; supporting Figure 2) [16] or plexiform neurofibroma (increased, hyperechogenic stroma between clearly discernible fascicles, and “bag of worms” appearance) were delineated in this teenage patient. A fascicular biopsy for further diagnostic purposes has not been performed to date due to her long-standing stable clinical and imaging course, but previewed in case of any change within the close monitoring. Malignant transformation of PNST in *NF2*-related schwannomatosis is exceedingly rare [28], especially without preceding radiation. Correspondingly, there was no conspicuous central necrosis, haemorrhage, peritumoral oedema, calcification, rich vascularization, and intratumoral lobulation [16] in the here presented case.

The pathogenic splice variant c.1123-2A > G, p.? affects the exons 11–15 of the *NF2* gene which, within the known genotype-phenotype correlation, has been associated with a milder disease course [29]. However, the young manifestation age in this girl argues against this. The importance of thorough phenotyping including detailed HRUS nerve imaging in *NF2*-related schwannomatosis, within the differential work-up of hypertrophic (mono)neuropathy [20], is corroborated by the here presented case report.

## Data Availability

The data that support the findings of this report are available upon request.

## Nomenclature

CSA: Cross-sectional area
Dist: Distal
HNST: Hybrid nerve sheath tumour
MRC: Medical research council
MRI: Magnetic resonance imaging
NF2: Neurofibromatosis type 2
PNST: Peripheral nerve sheath tumours
Prox: Proximal
SPACE: Sampling Perfection with Application optimized Contrast using different flip angle Evolution
STIR: Short-TI Inversion Recovery
TSE FS: Turbo-spin-echo fast saturation
US: Ultrasound

## Supporting Information

Supporting Figure 1: Axial, T2 TSE MRI (A), depicting a small, hyperintense vestibular schwannoma on the right side (white arrow) with significant contrast-enhancement on axial (B) as well as coronal (C) T1 postcontrast MRI.

Supporting Figure 2: For comparison to Figure 1 and 2, nerve ultrasound imaging of a patient with neurofibromatosis type 1 is shown, demonstrating a classic neurofibroma on axial imaging of the tibial nerve (A) and longitudinal imaging of the superficial peroneal nerve (B) (C).

Supporting Table 1: Nerve conduction study of upper extremities: signs of a motor more than sensory, predominantly axonal neuropathy for the right ulnar nerve, with normal findings on the contralateral side and for the median nerve (motor). Follow-up of the ulnar nerve showed no significant change.

Supporting Table 2: Nerve ultrasound measurements of peripheral nerves and cervical nerve roots (cross-sectional area (CSA, in mm^2^) or diameter (in mm)) at 3 different time points, compared to the 90th percentile of age-related normal values ^30^: Pronounced enlargement of the ulnar nerve from the distal upper to distal lower arm, as well as the cervical nerve root C5 and C6 on the right side and cervical nerve root C7 on the left side. In between these enlargements, normal size of the examined peripheral nerves and cervical nerve roots.

Supporting Videos 1–5: Videos depicting the right ulnar nerve in the axial plane at different levels from the wrist to the elbow, demonstrating a prominent, multifascicular, fusiform enlargement of the ulnar nerve, with a maximum extent in the proximal forearm.

Supporting Videos 6–7: Videos depicting the right brachial plexus in the axial plane, demonstrating a prominent, multifascicular, partly multinodular enlargement of the superior truncus and cervical nerve roots C5 and C6.
