# Supplementary material for: Distinct Imaging Features of Peripheral Nerve Sheath Tumours in NF2-Related Schwannomatosis: A Case Report
Source: Case Rep Neurol Med. 2025 Oct 9;2025:6923539. doi: 10.1155/crnm/6923539 (PMC12530925; doi:10.1155/crnm/6923539)
Supplement: Supporting Information — Additional supporting information can be found online in the Supporting Information section. [file 6923539.f1.zip › Supp_Table 1.docx]

**Supp Table 1:** Nerve conduction study of upper extremities: signs of a motor more than sensory, predominantly axonal neuropathy for the right ulnar nerve, with normal findings on the contralateral side and for the median nerve (motor). Follow-up of the ulnar nerve showed no significant change.

| **Ulnar nerve right / left** | | | | | | |
| --- | --- | --- | --- | --- | --- | --- |
| **Age (y)** | **Sens. Lat (ms)** | **Sens. Ampl. (uV) [Norm]** | **Sens. NCV (m/s) [Norm]** | **DML (ms) [Norm]** | **Mot. Ampl. (mV) [Norm]** | **Mot. NCV (m/s) [Norm]** |
| 10 | 2.2 / 1.7 | **4.4** / 18 [5.8] | **39** / 48 [44] | **3.5** / 2.3 [3.2] | **1.0** / 16.4 [4] | **12** / 55 [50] |
| 11 | 2.9 | **4.3** [5.8] | **41** [44] | **4** [3.2] | **0.8** [4] | **18** [50] |
| **Median nerve right** | | | | | | |
| 10 |  |  |  | 4.1 [4.6] | 13.5 [5] | 53 [50] |
| 11 | 3.1 [3.2] | 13.5 [6.9] | **37** [46] | 4.5 [4.6] | 13.7 [5] | 50 [50] |
| **F-Waves median nerve right** | | | | | | |
| 10 | 22 ms (Norm 31 ms), 10/10 | | | | | |
| 11 | 23.6 ms (Norm 31 ms), 10/10 | | | | | |
