# Supplementary material for: Distinct Imaging Features of Peripheral Nerve Sheath Tumours in NF2-Related Schwannomatosis: A Case Report
Source: Case Rep Neurol Med. 2025 Oct 9;2025:6923539. doi: 10.1155/crnm/6923539 (PMC12530925; doi:10.1155/crnm/6923539)
Supplement: Supporting Information — Additional supporting information can be found online in the Supporting Information section. [file 6923539.f1.zip › Supp_Table 2.docx]

**Table 2:** Nerve ultrasound measurements of peripheral nerves and cervical nerve roots (cross-sectional-area (CSA, in mm^2^) or diameter (in mm)) at 3 different time points, compared to the 90^th^ percentile of age-related normal values ^30^: Pronounced enlargement of the ulnar nerve from the distal upper to distal lower arm, as well as the cervical nerve root C5 and C6 on the right side and cervical nerve root C7 on the left side. In between these enlargements, normal size of the examined peripheral nerves and cervical nerve roots.

| **Nerve** | **Localization** | **CSA (mm^2^), right/left at age 10y** | **CSA (mm^2^), right/left at age 11y** | **CSA (mm^2^), right/left at age 12y** | **Normal value ^30^ (P90)** |
| --- | --- | --- | --- | --- | --- |
| **Ulnar** | Loge-de-Guyon | 4 | 5 | 4 |  |
|  | Wrist | **5-8** / 5 | 4 / 5 | 4 |  |
|  | Distal forearm | **8** / 4 | **8** | **8** |  |
|  | Mid forearm |  | **22** | **25** | 6.5 |
|  | Proximal forearm | **38** / 6 | **35-38** / 5 | **35-38** |  |
|  | Sulcus ulnaris | **7** / 3 | **8** / 4 | **7** | 5.5 |
|  | Distal upper arm |  | **15** / 5 | **18** |  |
|  | Mid upper arm | 4 / 3 | 4 | 5 | 7 |
|  | Proximal upper arm | 4 | 3 | 4 |  |
| **Median** | Wrist | 9 / 10 | 9 / 10 |  | 10 |
|  | Mid forearm | 5 / 5 | 5/ 3 |  | 7.5 |
|  | elbow | **9** / 7 | 7 / 7 |  | 8.5 |
|  | Mid upper arm | 8/ 8 | 8 / 8 |  | 10 |
|  | Proximal upper arm | 5 | 5 |  |  |
| **Radial** | Mid upper arm | 4 / 7 | 5 / 6 |  |  |
| **Radial superficial** | Proximal forearm | 1 / 1 | 1 / 1 |  | 2 |
| **Radial profundus** | Proximal forearm | 1 / 1 | 1 / 1 |  | 2.5 |
| **Tibial** | Knee | 17 / 17 | 17 / 18 |  | 28 |
|  | Distal lower leg | 6 / 6 | 8 / 7 |  | 9 |
| **Peroneal** | Knee | 4 / 5 | 6 / 7 |  | 6.5 |
| **Peroneal superficial** | Distal lower leg | 1 / 1 | 1 / 2 |  | 3 |
| **Sural** | Distal lower leg | 1 / 1 | 4 / 2 |  | 2.5 |
| **Vagal** |  | 3 |  |  |  |
| **Cervical root C5** | Diam (mm) prox | 2 | 2.2 / 2.5 | 2.3 / 2.5 | 2.7 |
|  | Diam (mm) dist | **4.5** | **4.1** / 3 | **5** / 2.6 |  |
| **Cervical root C6** | Diam (mm) prox | 2.5 | 2.9 / 2.8 | 2.5 | 4 |
|  | Diam (mm) dist | **6.1** | **6.5** | **5.4** |  |
| **Cervical root C7** | Diam (mm) prox | 3.5 / **8.2** | 3.3 / **7.7** | 3.4 / **7.7** |  |
| **Cervical root C7** | Diam (mm) dist | X / 2.7 | 3.6 / 2.4 | X / 3.9 |  |
| **Cervical root C7** | CSA prox |  | X / **48** |  |  |
