# Supplementary figures and images for: Distinct Imaging Features of Peripheral Nerve Sheath Tumours in NF2-Related Schwannomatosis: A Case Report
Source: Case Rep Neurol Med. 2025 Oct 9;2025:6923539. doi: 10.1155/crnm/6923539 (PMC12530925; doi:10.1155/crnm/6923539)

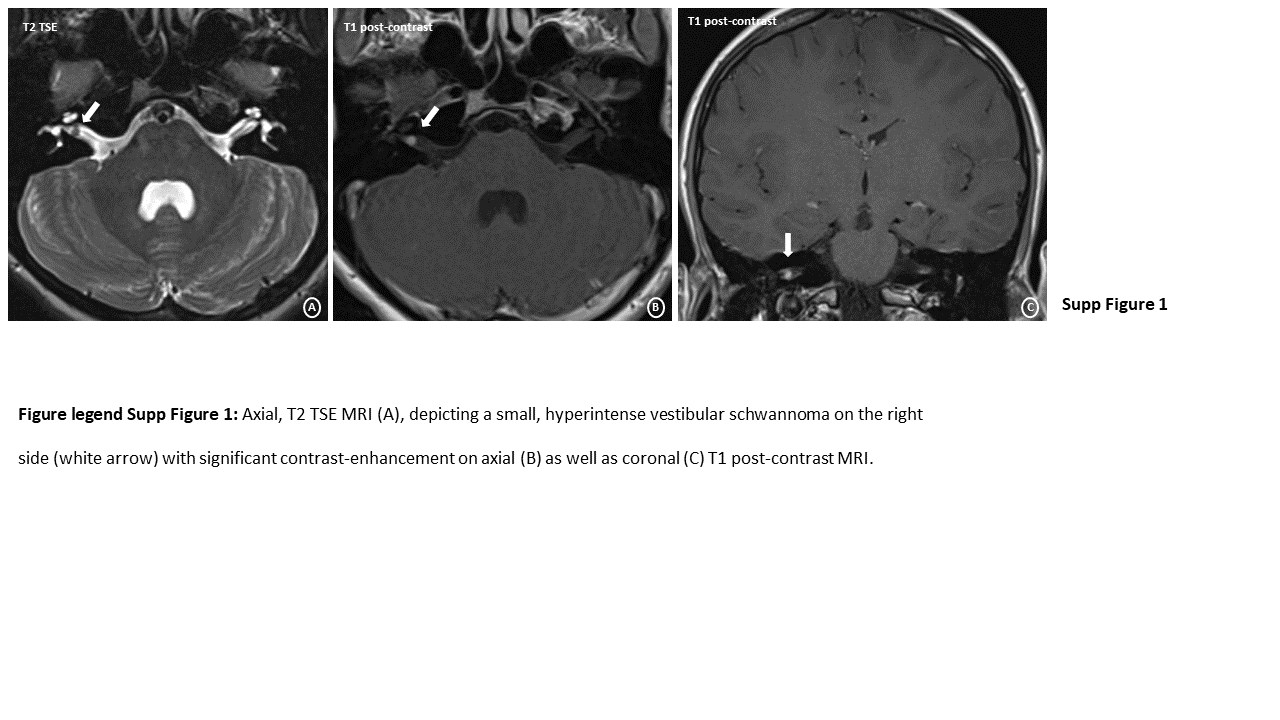

Supplement: Supporting Information — Additional supporting information can be found online in the Supporting Information section. [file 6923539.f1.zip › Supp_Figure_1.PNG]

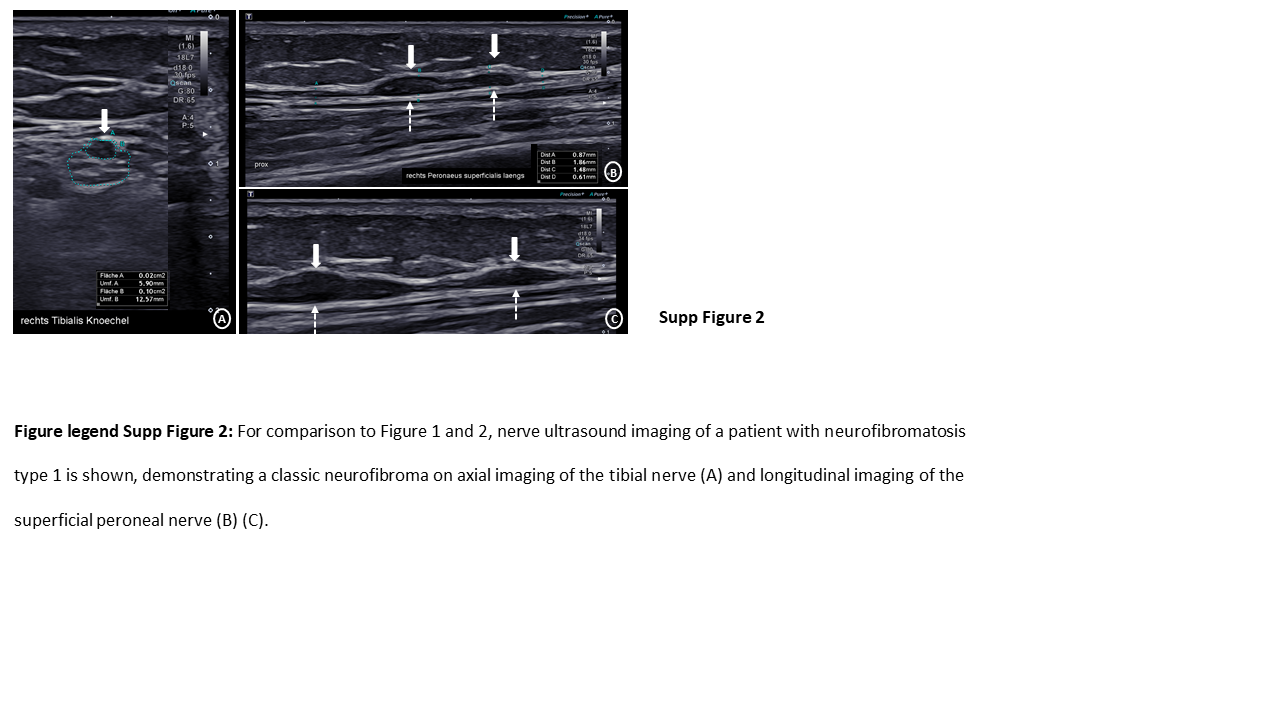

Supplement: Supporting Information — Additional supporting information can be found online in the Supporting Information section. [file 6923539.f1.zip › Supp_Figure_2.PNG]
